# Reduced approach disposition in familial risk for depression: Evidence from time-frequency alpha asymmetries

**DOI:** 10.1371/journal.pone.0307524

**Published:** 2024-07-24

**Authors:** Carola Dell’Acqua, Tania Moretta, Simone Messerotti Benvenuti

**Affiliations:** 1 Department of General Psychology, University of Padua, Padua, Italy; 2 Padova Neuroscience Center (PNC), University of Padua, Padua, Italy; 3 Hospital Psychology Unit, Padua University Hospital, Padua, Italy; Inner Mongolia University of Science and Technology, CHINA

## Abstract

Despite the promising role of alpha and delta power in reflecting reduced approach disposition in depression, to date, it is unclear whether these measures can be employed to identify at-risk individuals. Hence, the present study investigated affective disposition in 32 unaffected individuals with a family history of depression (23 F) and 30 individuals without a family history of depression (21 F) through a data-driven analysis of alpha and delta time-frequency power during the viewing of pleasant, neutral, and unpleasant pictures. Different patterns of posterior alpha asymmetry emerged within each group. Particularly, controls showed greater right posterior alpha desynchronization ~ 600 ms following emotional relative to neutral pictures presentation. Conversely, the group with a family history of depression showed greater posterior left alpha desynchronization only to unpleasant relative to neutral images in a later time window (> 900 ms). Hence, depression vulnerability seems to be characterized by a blunted reactivity to pleasant and delayed reactivity to unpleasant stimuli with a distinct posterior distribution relative to the controls. Finally, the two groups showed a comparable pattern of greater delta power to emotional relative to neutral cues. Overall, initial support was provided for the employment of time-frequency alpha power changes during affective processing in identifying blunted approach disposition in unaffected at-risk individuals.

## Introduction

It is estimated that approximately 322 million people are living with depression globally [1]. Depression affects psychological, physiological, and social functioning and is considered one of the leading causes of disease burden worldwide [1]. Given the pervasive nature of this condition, improving its early identification is of great importance. To this aim, the field of clinical psychophysiology is increasingly shifting its focus from the study of clinical depression correlates to the exploration of potential markers of this perilous condition [2]. One reliable risk condition is having a family history of depression, whereby individuals with a first-degree relative with a history of depression are three to five times more likely to develop depression themselves [3]. However, although the familial risk is well-established, it is still necessary to identify the mechanisms underlying this vulnerability.

Drawing from affective models of depression, it has been suggested that dysregulated reactivity to emotional cues could represent a potential marker for depression risk. For instance, coherently with the positive attenuation hypothesis, recent research suggests that vulnerability to depression appears to be mostly characterized by the hypoactivity of the approach-related motivational system, indexed by reduced emotional reactivity to appetitive cues [4–9]. Additionally, although findings are more mixed, psychophysiological research has recently suggested that depression and its risk might be characterized by reduced reactivity to unpleasant cues, indicating reduced withdrawal-related motivation [10–14]. The lack of reactivity to all emotional stimuli is aligned with the emotion context insensitivity hypothesis [ECI], which suggests that depression and its risk might be characterized by a blunted emotional reactivity in depression vulnerability [10, 15].

A psychophysiological measure reflecting the activity of the approach and withdrawal motivational systems is electroencephalographic [EEG] frontal alpha asymmetry [8–13 Hz] [16, 17]. This model is based on the well-established role of the left frontal activity in approach disposition, whereby reduced left relative to right frontal activity reflects a reduction in approach behaviors and increased withdrawal disposition. Given that alpha power is an inverse measure of the level of cortical activation, it has been widely employed to study the balance between the two motivational systems in the brain in individuals with depression [18]. An asymmetric pattern of frontal alpha activity, with increased alpha in the left frontal lobe compared to the right, is believed to reflect the hypoactivation of the approach-related motivation system and has long been considered a potential biomarker for depression in resting-state conditions [19]. Resting-state posterior asymmetry was also examined, and reduced right parietal activity [i.e., greater alpha power] was observed in depression [20–22] and in at-risk samples [21, 23]. Several studies have also observed greater overall posterior alpha in adults and adolescents with depression [24–27].

However, following the recent criticism about the reliability of resting-state alpha asymmetry as a viable biomarker of depression [28], the capability model has suggested that dysregulated affective disposition might be more evident in response to emotional contexts than at rest [29]. To date, a limited number of studies on EEG alpha asymmetry during an emotional task have observed lower relative left frontal activity in response to pleasant and unpleasant contexts in individuals with depressive symptoms [7, 29]. Another recent study that explored time-frequency alpha power with a data-driven approach has evidenced a smaller alpha desynchronization in bilateral frontal and right centro-parietal regions to pleasant images in dysphoria [30]. Given that the right posterior cortex has a role in emotional processing [31–33], these results were interpreted as an under-engagement and reduced reactivity to pleasant images in dysphoria. The method employed by the latter study is advantageous as it allowed the exploration of the time course and localization of emotional reactivity rather than averaging brain activity over several seconds across anterior scalp location.

More recently, time-frequency delta power [< 3 Hz] has been studied as an index of approach disposition [34]. Delta oscillations typically increase in centroparietal regions in response to emotional stimuli relative to neutral ones and are thought to play a role in monitoring motivational salience and identifying appetitive cues in the environment [35–41]. Previous studies have observed reduced time-frequency delta power to pleasant or rewarding stimuli in clinical depression [42, 43], in dysphoria [4], and prospectively predict depression onset [44].

Despite the promising role of alpha power asymmetries and delta power during the viewing of pleasant stimuli in reflecting reduced approach disposition in depression and its risk, to date, these measures have never been investigated in individuals with a familial risk for depression. However, to understand whether blunted approach disposition can be considered a risk factor and not merely a manifestation of current depression, it is necessary to examine these processes in high-risk individuals who have never presented with clinically significant symptoms. To this end, the objective of the present study was to examine affective disposition in unaffected individuals with a family history of depression through the analysis of alpha and delta time-frequency patterns during the viewing of emotional pictures from the International Affective Picture System [IAPS, [45]]. Building on previous evidence on the functional correlates of alpha and delta bands, the group with a family history for depression was expected to show a hypoactivity of the approach-related motivational system in the brain, indexed by reduced alpha desynchronization in the left frontal and right parietal sites and reduced centro-parietal delta to pleasant [but not neutral and unpleasant] images relative to controls. In addition, although findings are more mixed, in line with the ECI hypothesis, an hypoactivity of the withdrawal motivational system in the brain could be expected in the group with a family history of depression, indexed by reduced alpha desynchronization in right anterior sites and reduced centro-parietal delta power to unpleasant images.

## Materials and methods

### Participants

The present study is a secondary analysis of EEG data collected during a passive viewing paradigm in a sample of 62 Italian university students at the University of Padua, Italy (see [46] for further details on the sample). This secondary analysis does not overlap in terms of scope or analysis with the first study, which examined emotion-related attentional processes in familial risk for depression. As in detailed in our previous work [46], the Beck Depression Inventory-II (BDI-II, [47, 48]) and the module A of the Structured Clinical Interview for DSM-V (SCID 5-CV; [47, 48]) were employed to assess the presence of current and past depressive symptoms. The Family History Screen (FHS, [49]) was administered to assess the presence of current or past depression and/or other psychopathological conditions in first-degree relatives. All the enrolled participants had a BDI-II score lower than 12 and did not meet the diagnostic criteria for a major depression episode, persistent depressive disorder, or bipolar disorder. Furthermore, based on the FHS, 32 participants with at least one first-degree relative with a history of depression were assigned to the group with a family history for depression (23 females; mean age ± standard deviation (SD) = 21.9 ± 3.3; mean BDI-II score ± SD = 5.4 ± 3.5), while 30 participants whose first-degree relatives did not have a family history of any psychiatric disorder were assigned to the group without a family history for depression (21 females; mean age ± standard deviation (SD) = 21.0 ± 3.2; mean BDI-II score ± SD = 4.8 ± 3.1). The two groups were matched in terms of sex, age, and years of education (see [46]). All participants were white. Participants were compensated for their participation (13 euros per visit). Written informed consent was obtained from all participants. The research was conducted in compliance with the World Medical Association Declaration of Helsinki on research on human subjects and was approved by the Ethical Committee of Psychological Research, Area 17, University of Padova (prot. no. 3712).

### Procedure

Before the experimental session in the laboratory, participants were required to avoid alcohol intake the day before and to avoid caffeine and nicotine the same day of the visit. Written informed consent was obtained from all participants, then participants were administered the ad-hoc anamnestic interview, the mood episode module (module A) of the SCID-5-CV, the FHS, and the BDI-II. Then, participants were seated on a comfortable chair in a dimly lit, sound-attenuated room. After electrode placement and a three-minute resting-state period, six practice trials including two pleasant, two neutral, and two unpleasant pictures were provided. Then, participants underwent the emotional passive viewing task. Following the task, participants were required to evaluate the valence and arousal of 36 pictures (12 for each category) using the Self-Assessment Manikin scale (SAM, [50], an assessment tool that directly measures valence and arousal associated with a person’s affective reaction to the stimuli on a non-verbal 9-point scale (for details on self-report data see [46]). The entire procedure took approximately 90 min.

### Psychological evaluation

A trained doctoral-level clinical psychologist conducted the interview to assess the presence of depressive disorders in participants and their first-degree relatives. The Structured Clinical Interview for DSM-5 (SCID-5-CV; [47, 48]) was employed to exclude individuals with a current mood disorder (major depressive episode, persistent depressive disorder, or bipolar disorder). As in our previous work ([46]), the Italian translation of the FHS ([49]) was used to assess the presence of family psychiatric conditions in first-degree relatives (i.e., biological parents, and siblings). members. An affirmative answer to item 8 (“*Did one of your parents or siblings ever have a period of feeling sad*, *blue*, *or depressed for most of the time for at least two weeks*? *Please answer by reporting the member of your family who experienced these feelings without including time of physical illness or mourning after a death*”) and/or to item 9 (“*Did one of your parents or siblings ever have a period (at least two weeks) of feeling quite tired*, *having less energy*, *or not caring about their usual activities*? *Please answer by reporting the member of your family who experienced these feelings without including time of physical illness or mourning after a death*”) was considered as indicative of a first-degree relative with a history of depression ([46]).

Depressive symptoms in the past two weeks were assessed using the BDI-II. The BDI-II is a self-report measure composed of 21 items, each with a four-point Likert scale and scores ranging from 0 to 63, with higher scores indicating greater depressive symptoms. For this study, Cronbach’s alpha was α  =  .91 indicating high internal consistency.

### Experimental task

The picture viewing task comprised a total of 72 color pictures selected from the International Affective Picture System (IAPS, [45]: 24 pleasant (i.e., erotic couples, sports), 24 neutral (i.e., neutral faces, household objects), and 24 unpleasant (i.e., attacking humans and animals). Pleasant and unpleasant pictures were matched for normative arousal ratings and were significantly higher than neutral pictures (*p* < .001). All pictures were presented for 6000 ms in semi-random order across three blocks of 24 trials (i.e., no more than one stimulus in the same emotional condition had to be shown consecutively). Each picture was preceded by a 3000 ms interval where a white fixation cross was placed centrally on a grey screen. Participants were required to keep their gaze on the center of the screen. Picture presentation was followed by a variable intertrial interval of 6000–8000 ms, during which a white fixation cross was presented. Picture presentation lasted for 6000 ms as the project included the registration of electrocardiographic activity to analyze cardiac deceleration.

### EEG recording and data processing

The EEG was recorded using a 32-channel system (ANT Neuro, Enschede, Netherlands) referenced online to CPz with a sampling rate of 1000 Hz. Both vertical and horizontal electrooculograms (EOGs) were recorded using a bipolar montage to register eye movements and eye blinks. The electrode pairs were placed at the supra- and suborbit of the right eye and at the external canthi of the eyes, respectively. Electrode impedance was kept below 10 kΩ.

Offline EEG data processing was conducted using EEGLAB [51] and Brainstorm [52]. The EEG signal was downsampled to 500 Hz and re-referenced to the average mastoid electrodes and filtered from 0.03 to 30 Hz. Blinks were corrected using independent component analysis (ICA). Epochs from 3000 ms before until 3000 ms after picture onset were extracted. Each epoch was baseline-corrected by subtracting the mean pre-stimulus voltage between −250 ms and −50 ms. Segments that contained residual artifacts exceeding ±70 μV (peak-to-peak) were excluded.

Time-frequency analysis was conducted using Morlet wavelet transformation on individual trials for each 1-Hz frequency bin between 1 and 30 Hz, using a mother wavelet at 1 Hz with 3-s time resolution (as calculated by the full width at half maximum, FWHM). Time-frequency decompositions were then averaged for each participant and emotional condition, and the event-related spectral perturbation (ERSP) was computed as the change in power expressed in decibels (dB) relative to the baseline (−900 to −400 ms) in each frequency bin at each time point (i.e., baseline normalization). Then, data were grand averaged across each group for each emotional condition.

### Statistical analyses

To compute differences within- and between groups in event-related delta and alpha power, a cluster-based permutation approach was adopted as implemented by the FieldTrip toolbox [53, 54]. This is an open-access software that performs statistical correction for multiple comparisons by identifying spatio-temporal event-related differences and tests their statistical significance using baseline values derived from Monte-Carlo simulated data [53]. Details of this statistical approach can be found in previous publications from our group [4, 42, 55, 56] and other groups [57–61]. Briefly, cluster-based statistics rely on a data-driven distribution of the test statistic and the initial non-permuted test statistic is compared against the shuffled distribution and a cluster-level correction was applied [62]. In the present study, to obtain the null hypothesis distribution, data were shuffled 2000 times. The cluster-corrected *p*-value was calculated by comparing the statistics of the proportion of clusters in the null distribution that surpassed the one observed in each significant cluster within the non-shuffled data. Clusters of at least two electrodes and with a *p*_corr_ < .05 were considered statistically significant. This method allows to reliably control over type I error rate arising from multiple comparisons across electrodes and time points ([53, 54]. Cluster-based repeated measures ANOVAs were conducted to test within-group differences in event-related delta (1–3 Hz) and alpha (9–14 Hz) power changes between emotional categories (i.e., pleasant, unpleasant, and neutral). Two-tailed independent samples *t*-tests were conducted to test between-group (i.e., with a family history of depression, without a family history of depression) differences within each emotional category. To examine the internal consistency of the significant time-frequency clusters, split-half reliability was computed by taking the correlation between even and odd trials for each category (pleasant, neutral, unpleasant) and then adjusting with the Spearman-Brown prediction formula.

## Results

### Delta power (1–3 Hz)

#### Differences among emotional categories in event-related delta power

**Fig 1:**
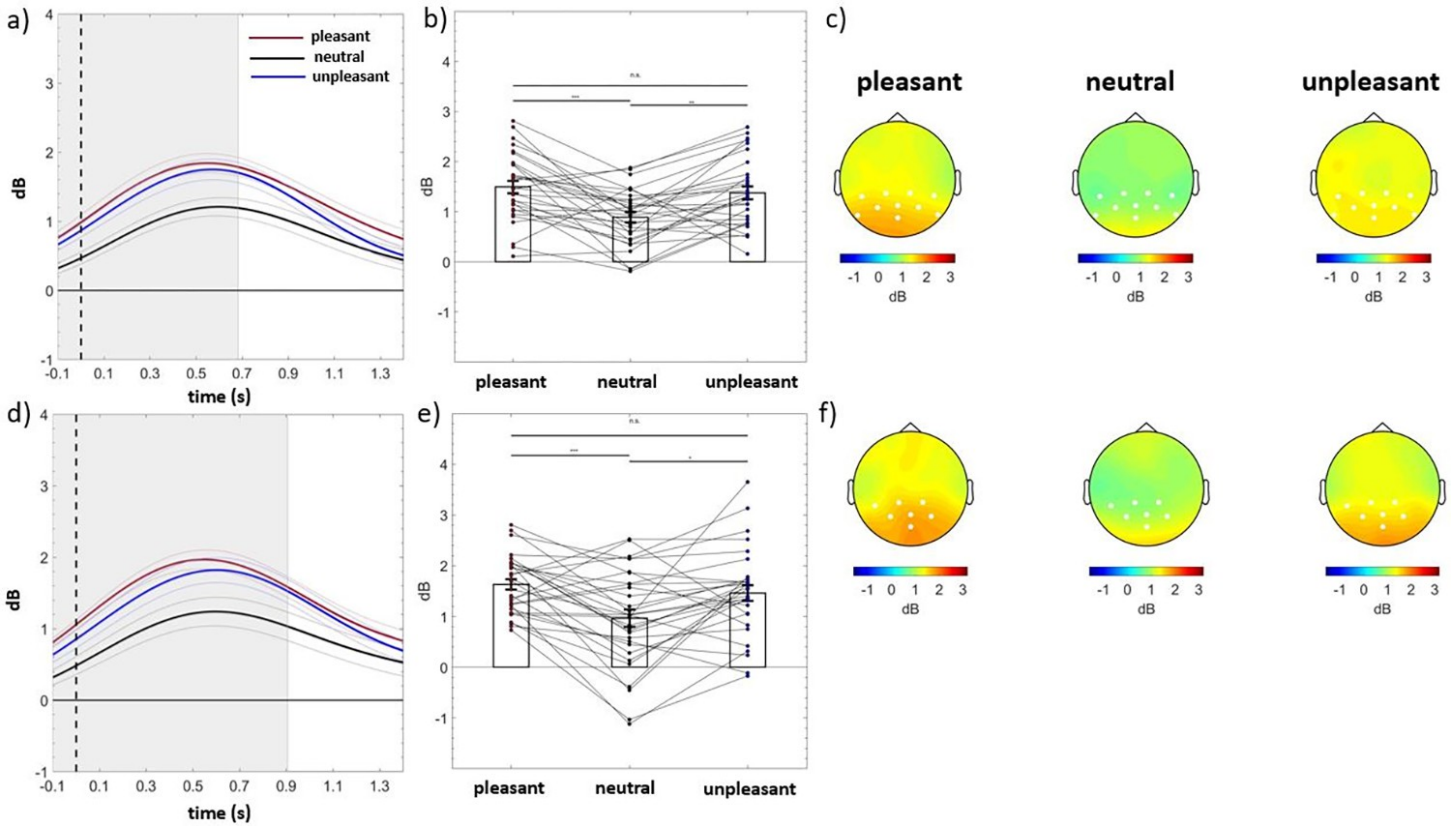
Time-frequency delta power. [Panels a and d] Time course of event-related delta power of individuals without a family history of depression (above) and with a family history of depression (below) averaged over the significant electrodes for pleasant (red line), neutral (black line), and unpleasant (blue line) conditions. Shaded areas represent ± standard error of the mean (SEM). [Panels b and e] Mean event-related delta power of each participant in the group without a family history of depression (above) and with a family history of depression (below) averaged over the significant electrodes and time points for pleasant, neutral, and unpleasant conditions. Each circle represents one participant; colored frames represent the mean event-related delta power across all participants and the solid black lines represent ± SEM. *p < .05; ***p < .001. [Panels c and f] Topography of the mean event-related delta power of individuals without a family history of depression (above) and with a family history of depression (below) for pleasant, neutral, and unpleasant conditions.

A significant centro-parietal cluster emerged (electrodes = CP5 CP1 CP2 CP6 P7 P3 PZ P4 P8 POZ) in the group without a family history for depression (cluster *F*-valuemax = 60427.14, *p*corr < .001, time window = -100 to 682 ms) and in the group with a family history for depression (electrodes = CP5 CP1 CP2 P3 PZ P4 POZ; cluster *F*-valuemax = 48259.94, *p*corr = .001, time window = -100 to 906 ms), as shown in panels a, b, c, and d, e, f of Fig 1, respectively. Both groups showed significantly greater delta to pleasant and unpleasant stimuli relative to neutral ones (all *p*s ≤ .013; Fig 1). Split-half reliability, which was computed by taking the correlation between the average of even and odd trials and then adjusting with the Spearman-Brown prediction formula, showed that the reliability estimates of time-frequency delta power were low [cluster within the group without familial risk for depression: pleasant trials, *r* (*p*-value) = .30 (.11), neutral trials, *r* (*p*-value) = -.02 (.90), unpleasant trials, *r* (*p*-value) = .31 (.09); cluster within the group with familial risk for depression: pleasant trials, *r* (*p*-value) = -.01 (.97), neutral trials, *r* (*p*-value) = .44 (.01), unpleasant trials, *r* (*p*-value) = .39 (.03)].

#### Differences between groups in event-related delta power for each emotional category

No significant cluster for the difference between the groups within each emotional condition in delta power emerged.

### Alpha power (9–14 Hz)

#### Differences among emotional categories in event-related alpha power

**Fig 2:**
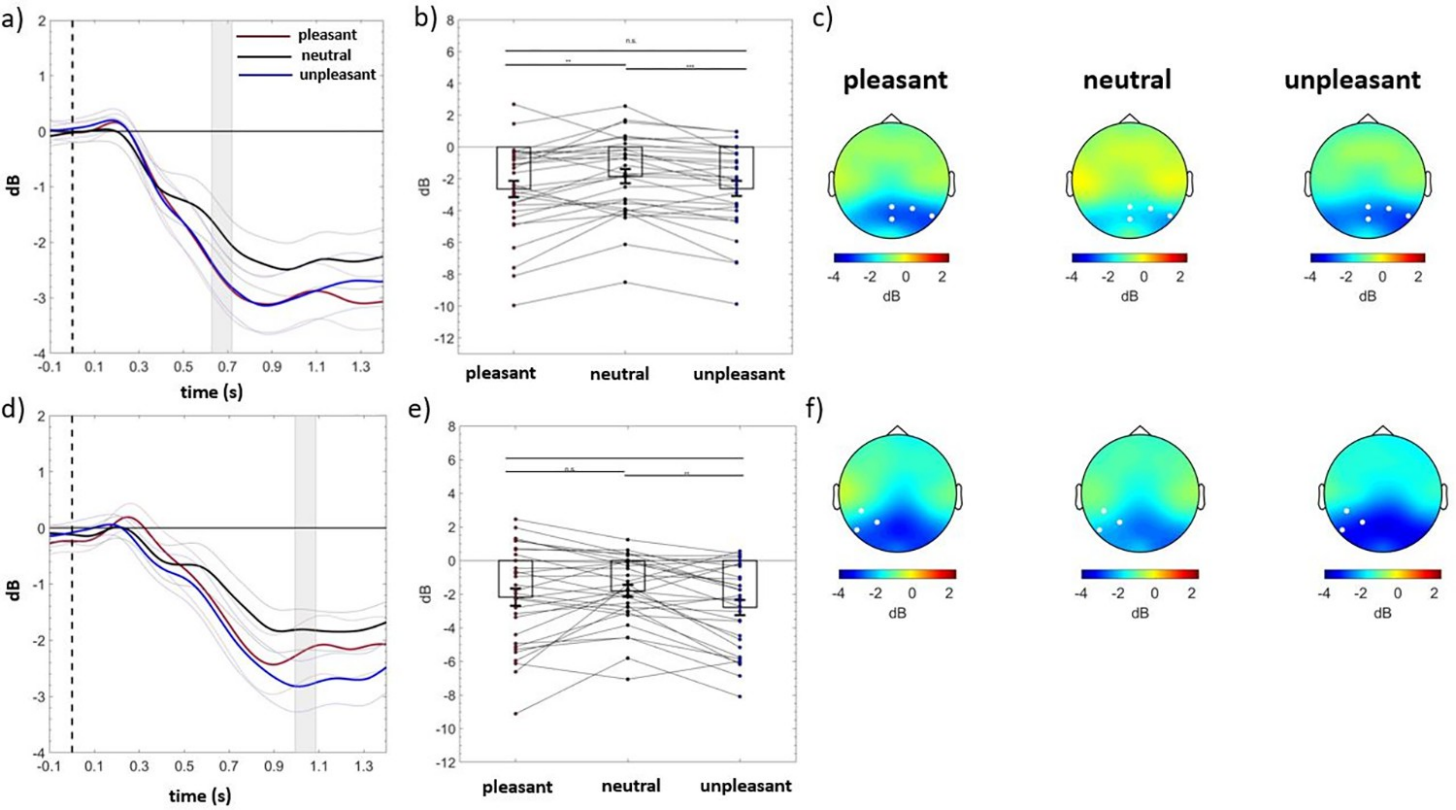
Time-frequency alpha power. [Panels a and d] Time course of event-related alpha power of individuals without a family history of depression (above) and with a family history of depression (below) averaged over the significant electrodes for pleasant (red line), neutral (black line), and unpleasant (blue line) conditions. Shaded areas represent ± standard error of the mean (SEM). [Panels b and e] Mean event-related alpha power of each participant in the group without a family history of depression (above) and with a family history of depression (below) averaged over the significant electrodes and time points for pleasant, neutral, and unpleasant conditions. Each circle represents one participant; colored frames represent the mean event-related alpha power across all participants and the solid black lines represent ± SEM. *p < .05; ***p < .001. [Panels c and f] Topography of the mean event-related alpha power of individuals without a family history of depression (above) and with a family history of depression (below) for pleasant, neutral, and unpleasant conditions.

A significant right parietal cluster emerged (electrodes = PZ P4 P8 POZ) in the group without a family history of depression (cluster *F*-valuemax = 2680.50, *p*corr = .035, time window = 628 to 718 ms). Particularly, in this group, alpha power was reduced in response to pleasant and unpleasant images relative to neutral ones, and no difference between the two emotional categories emerged (unpleasant vs. neutral, *p* < .001; unpleasant vs. pleasant, *p* = .566 pleasant vs. neutral, *p* = .009). Instead, within the group with a family history of depression, a left parietal cluster emerged (electrodes = CP5 P7 P3; cluster *F*-valuemax = 2059.52, *p*corr = .048, time window = 994 to 1086 ms), as shown in panel a, b, c, and d, e, f of Fig 2, respectively. Particularly, within this group, alpha power was significantly reduced in response to unpleasant relative to neutral and pleasant trials and no difference emerged between pleasant and neutral images (unpleasant vs. neutral, *p* = .003; unpleasant vs. pleasant, *p* = .034 pleasant vs. neutral, *p* = .691). Split-half reliability, which was computed by taking the correlation between the average of even and odd trials and then adjusting with the Spearman-Brown prediction formula, showed that the reliability estimates of time-frequency alpha power were moderate-to-high [cluster within the group without familial risk for depression: pleasant trials, *r* (*p*-value) = .68 (< .001), neutral trials, *r* (*p*-value) = .77 (< .001), unpleasant trials, *r* (*p*-value) = .70 (< .001); cluster within the group with familial risk for depression: pleasant trials, *r* (*p*-value) = .77 (< .001), neutral trials, *r* (*p*-value) = .59 (< .001), unpleasant trials, *r* (*p*-value) = .86 (< .001)].

#### Differences between groups in event-related alpha power for each emotional category

No significant cluster for the difference between the groups within each emotional condition in alpha power emerged.

## Discussion

This work aimed at investigating affective processing in a population at-risk to develop depression, namely unaffected individuals with a family history of depression, through the analysis of alpha and delta time-frequency dynamics during the viewing of pleasant, neutral, and unpleasant pictures from the International Affective Picture System (IAPS, [45]). Based on previous evidence, individuals with a familial risk for depression were expected to show reduced reactivity to pleasant images, indexed by reduced alpha desynchronization in the left frontal and right parietal sites and reduced centro-parietal delta to pleasant (but not neutral and unpleasant) images relative to controls.

With respect to time-frequency alpha power, partly in line with the hypothesis, the two groups showed distinct patterns of event-related activation to arousing emotional stimuli relative to neutral ones at the within-subjects level. Specifically, the group without familial risk for depression showed greater alpha desynchronization (i.e., reduced alpha power) in response to pleasant and unpleasant relative to neutral images, indicating greater cortical activation during the processing of affective stimuli. On the contrary, in line with previous evidence on other at-risk samples (i.e., dysphoria, [30]), the group with familial risk for depression showed greater alpha desynchronization only to unpleasant (and not to pleasant) relative to neutral ones. Hence, despite individuals with familial risk for depression being currently free from depressive symptoms, they did not show greater cortical activation to highly arousing pleasant images but, instead, showed a similar pattern of activation for pleasant and neutral cues. This result provides support to the positive attenuation hypothesis, suggesting that familial risk for depression might be characterized by reduced approach disposition. Regarding the localization of these effects, the control group showed greater cortical activation to emotional images in a right posterior cluster, which is in line with the literature suggesting that right posterior regions might be implicated in the processing of highly arousing cues [63]. The at-risk sample did not show greater right cortical activation while processing emotional images but, instead, their emotional processing seems to be steered by left posterior cortical activity. Regarding the timing of the observed effects, alpha power clusters of activity associated with affective processing occurred after 500 ms post-image presentation in both groups, supporting the hypothesis that affective modulation occurs later in the processing of visual salient cues [64]. Importantly, although unpleasant images elicited emotional reactivity in the group with familial risk for depression, this effect was significant in a later time window (~ 900 ms), suggesting that the processing of unpleasant cues might be delayed in this at-risk group. Thus, from these results, it could be hypothesized that individuals vulnerable to depression show an initial blunted reactivity to all emotional stimuli and only a late engagement to unpleasant ones. However, contrary to what was hypothesized, no between-group differences emerged in alpha power changes. The observed time-frequency alpha patterns in unaffected individuals with a family history of depression might represent an electrocortical indicator of depression risk, without the confounding effect of the presence of subclinical or clinical depressive symptoms. Considering that this was the first study looking at time-frequency alpha activity during emotional processing in a group of unaffected individuals with a family history of depression with a data-driven robust statistical method, further research is needed to clarify whether this differential pattern of alpha activity might constitute an indicator of depression risk. Of note, the split-half reliability of alpha power measures was moderate-to-high, suggesting that the signal-to-noise ratio was adequate.

The exploration of the above-described effects in terms of localization and timing was achieved through the employment of a data-driven approach to time-frequency EEG data, which made it possible to go beyond the typically explored frontal alpha asymmetry by examining significant clusters of activity across the whole brain and time points. Future studies with this robust statistical approach are warranted to better parse patterns of altered neural activity associated with affective dysregulation in both depression and its risk.

Regarding time-frequency delta power changes, in line with previous studies [4, 41], both groups showed a within-group pattern of greater delta power to highly-arousing (pleasant and unpleasant) relative to neutral pictures, confirming the role of delta power in affective modulation. However, contrary to what was hypothesized based on previous findings in other at-risk samples [e.g., 41]), no group differences emerged in time-frequency delta pattern changes. This finding suggests that the reduced delta power to appetitive cues might be a correlate of subclinical and clinical symptoms, but it might not be a reliable early indicator of vulnerability, at least in the present sample with a familial risk for depression. However, the split-half reliability of delta clusters was low, indicating that it likely requires more than 24 trials for each category to consistently capture delta power changes and enhance the signal-to-noise ratio of this measure.

The present findings should be interpreted in light of several limitations. First, the assessment of family history for depression was not directly based on clinical interviews with the family members but rather relied on self-reported interviews with the participants. Consequently, it may be that some participants may have lacked awareness of any instances of depression among their relatives, particularly if that occurred before the participants reached a certain age. Moreover, although the two groups did not differ in terms of sex, most of the participants belonged to the female sex and were White university students. Hence, considering also that this work has been conducted as a first hypothesis testing, larger confirmatory studies with more diverse samples should be designed. Also, although the emotional passive viewing task is a valid and widely used paradigm to study affective processing [65, 66] future studies that include specific experimental manipulations during the exposure to affective images are warranted to better identify neural pathways to depression. Moreover, while participants did not disclose any psychiatric diagnoses during the anamnestic interview, it is important to note that the potential presence of such conditions cannot be entirely excluded. This is because the utilization of only Module A of the SCID-5-CV limited our ability to thoroughly explore whether participants may meet criteria for psychiatric conditions other than depressive or bipolar disorders. Finally, the administration of module A of the SCID-5-CV interview by only one Psychologist did not allow us to evaluate inter-rater reliability. However, the SCID- 5-CV has excellent inter-rater reliability for most disorders [67].

Notwithstanding these limitations, the present study was the first attempt to explore affective processing in a sample of individuals with a family history of depression through a time-frequency data-driven approach. From the presented findings, vulnerability to depression seems to be characterized by a blunted reactivity to pleasant stimuli and a delayed reactivity to unpleasant stimuli with a distinct posterior distribution relative to the control group. Taken together, reduced approach disposition seems to characterize even unaffected individuals with a higher risk of developing depression, and alpha power changes during affective processing might be a valuable measure to identify those at risk for depression early, even in the absence of depressive symptoms.

## Supporting information

S1 Fig Time-frequency plots of spectral power on the difference between the pleasant and neutral (left) and the unpleasant and neutral (right) conditions for the group without a family history for depression in the significant cluster that emerged in this group (Pz, P4, P8, POz).
(TIF)

S2 Fig Time-frequency plots of spectral power on the difference between the pleasant and neutral (left) and the unpleasant and neutral (right) conditions for the group with a family history for depression in the significant cluster that emerged in this group (CP5, P7, P3).
(TIF)
