# Supplementary figures and images for: Reduced approach disposition in familial risk for depression: Evidence from time-frequency alpha asymmetries
Source: PLoS One. 2024 Jul 24;19(7):e0307524. doi: 10.1371/journal.pone.0307524 (PMC11268641; doi:10.1371/journal.pone.0307524)

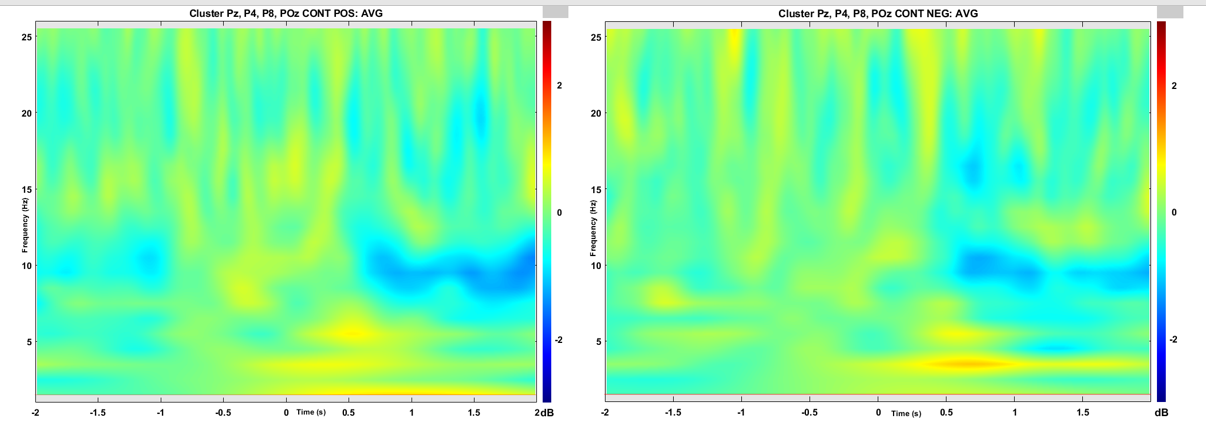

Supplement: S1 Fig — Time-frequency plots of spectral power on the difference between the pleasant and neutral (left) and the unpleasant and neutral (right) conditions for the group without a family history for depression in the significant cluster that emerged in this group (Pz, P4, P8, POz). (TIF) [file pone.0307524.s001.tif]

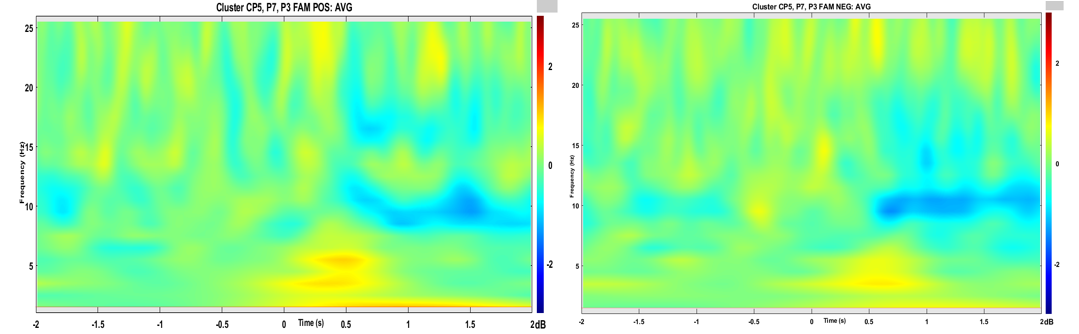

Supplement: S2 Fig — Time-frequency plots of spectral power on the difference between the pleasant and neutral (left) and the unpleasant and neutral (right) conditions for the group with a family history for depression in the significant cluster that emerged in this group (CP5, P7, P3). (TIF) [file pone.0307524.s002.tif]
